# The effect of early measles vaccination at 4.5 months of age on growth at 9 and 24 months of age in a randomized trial in Guinea-Bissau

**DOI:** 10.1186/s12887-016-0738-z

**Published:** 2016-12-03

**Authors:** S. M. Rasmussen, S. Biering-Sørensen, S. Byberg, A. Andersen, M. Bjerregaard-Andersen, A. Rodrigues, C. S. Benn, C. L. Martins, P. Aaby

**Affiliations:** 1 Bandim Health Project, Indepth Network, Apartado 861, 1004 Bissau Codex, Guinea-Bissau; 2 Bandim Health Project, Research Center for Vitamins and Vaccines (CVIVA), Statens Serum Institut, Artillerivej 5, 2300 Copenhagen S, Denmark; 3 OPEN, Odense Patient data Explorative Network, Odense University Hospital/Institute of Clinical Research, University of Southern Denmark, Odense, Denmark; 4 Department of Endocrinology, Odense University Hospital, Institute of Clinical Research, University of Southern Denmark, Odense, Denmark

**Keywords:** Early measles vaccination, Growth, Non-specific effects, Sex-differential effects, Neonatal vitamin A supplementation, Season

## Abstract

**Background:**

Providing an early, additional measles vaccine (MV) at 4.5 months of age has been shown to reduce child mortality in low-income countries. We studied the effects on growth at 9 and 24 months of age.

**Methods:**

A randomized controlled trial was conducted in Guinea-Bissau from 2003–2007 including 6,648 children. Children were randomized 1:1:1 to receive Edmonston-Zagreb measles vaccine at 4.5 and 9 months of age (group A), no vaccine at 4.5 months and Edmonston-Zagreb measles vaccine at 9 months (group B), or no vaccine at 4.5 months and Schwarz measles vaccine at 9 months (group C) Data on anthropometrics were obtained at enrolment at 4.5 months of age and again at 9 and 24 months of age. Analyses were stratified by sex, season of enrolment, and neonatal vitamin A supplementation (NVAS) status, as all these factors have been shown to modify the effect of early MV on mortality.

**Results:**

Overall there was no effect of early MV on anthropometry at 9 months. At 24 months children who had received early MV had a significantly larger mid-upper-arm-circumference (MUAC/in cm) (Difference = 0.08; 95% CI (0.02;0.14)) compared with children in the control group; this effect was most pronounced among girls (0.12 (0.03;0.20)). The effect of early MV on MUAC remained significant in the dry season and in girls who received placebo rather than NVAS.

**Conclusion:**

Early MV was associated with a larger MUAC particularly in girls. These results indicate that a two-dose measles vaccination schedule might not only reduce child mortality but also improve growth.

**Trial registration:**

ClinicalTrials.gov NCT00168558. Registered September 9, 2005, retrospectively registered

**Electronic supplementary material:**

The online version of this article (doi:10.1186/s12887-016-0738-z) contains supplementary material, which is available to authorized users.

## Background

When the measles vaccine (MV) was introduced in high mortality areas in Africa in the 1970s, studies reported mortality declines of 40% or more [1–6]. A decrease in mortality of this magnitude cannot be explained merely by prevention of measles cases and its long-term consequences, since measles deaths accounts for an estimated 10% of all deaths among under-5-years-olds [7]. Thus it has been suggested that MV may have beneficial heterologous or non-specific effects on the susceptibility to infectious diseases other than measles [3, 5, 8].

In low-income countries, the World Health Organization (WHO) recommends that children are vaccinated against measles at 9 months of age, to ensure protection as early in life as possible and at the same time minimizing interference from maternal antibodies (MA) [9]. In 2003–2007, the Bandim Health Project in Guinea-Bissau conducted a large randomized trial, the “Early MV trial”, to explore the potential benefits of providing an additional early MV [8]. In this trial children were randomized to receive two doses of MV at 4.5 months and at 9 months or only one dose of MV at 9 months of age [9]. The study found a beneficial effect of the two-dose regimen on mortality and hospitalizations [8, 10]. In the *per-protocol* analysis the mortality rate ratio (MRR) of children who received two doses of MV compared with one dose only was 0.70 (95% CI 0.52;0.94) between 4.5 and 36 months of age. The effect was significant for girls, when stratifying by sex. A large proportion (57%) of the children enrolled in the Early MV trial had previously participated in a trial of neonatal vitamin A supplementation at birth (NVAS) [11], [12]. Interestingly, NVAS seemed to modify the effect of early MV [8, 10]; early MV was not associated with mortality reductions in children who received NVAS, but only children who did not receive NVAS. The effect of an early MV on hospitalizations was also explored within this study, and early MV was found to reduce the rates of hospital admissions, especially admissions for respiratory infections [10]. Dry (December-May) vs. rainy (June-November) season were also found to influence the effect of MV on mortality [8]. The aim of the present study was to explore the effect of early MV on growth within the children enrolled in the “Early MV trial”. We pre-specified that we would explore whether sex and (NVAS), along with season, modified the effect of early MV on growth.

## Methods

### Setting

The early MV trial was conducted from 2003–2007 in Bissau, the capital of Guinea-Bissau, where the Bandim Health Project (BHP) has maintained a health and demographic surveillance system (HDSS) since 1978. The HDSS catchment area covers an area of 102,000 inhabitants divided into six different districts. All children less than three years of age are visited every three months at their home and various information is collected, including information on family conditions, hospital admissions, sickness and vital status [8]. At time of study, the routine vaccination schedule was BCG and oral polio vaccine (OPV) at birth, diphtheria, tetanus, pertussis (DTP) + OPV at 6,10 and 14 weeks and measles and yellow fever vaccines at 9 months of age. [8] The infant mortality rate was 138 per 1,000 live births, and the under-five mortality rate was 223 per 1,000 live births, the main causes of deaths being malaria, acute respiratory infections and diarrhea. An estimated 4% of the children were malnourished and 19% suffered from moderate malnutrition [13]. There was a measles outbreak in the early phase of the trial, from October 2003 to May 2004; 100 participants were diagnosed with measles. Apart from that, there was no circulating measles [8].

In the present study, children 4.5 to 7 months of age, residing within the study area were invited to participate. Children were only included if a minimum of 4 weeks since the third diphtheria, tetanus, pertussis (DTP) vaccine had passed. Oral and written explanations of the study were provided to the mother/guardian. Mothers/guardians willing to let their children participate signed a consent form by signature or fingerprint if illiterate. The children were individually randomized 1:1:1 into three groups; the first group received early Edmonston-Zagreb MV at 4.5 months of age and again at 9 months of age (Group A), the second group received no vaccine at 4.5 months and Edmonston-Zagreb MV at 9 months (Group B), and the third group received no vaccine at 4.5 months and Schwarz MV at 9 months (Group C).

For ethical reasons, no placebo was given in groups B and C at 4.5 months. If the mother moved away from the study area with the child and had received a placebo vaccine at 4.5 months, we feared that the mother would mistakenly think the child was already measles vaccinated and therefore not take the child to measles vaccination at 9 months. The staff assessing outcomes were unaware of the child’s vaccination status and were instructed not to ask the mother if the child had received an early MV or not.

The study design has been described in detail elsewhere [8]. Children included in the study visited the health center on three occasions: at inclusion, at 9 months and 24 months of age. The children in group B and C also visited the health center at 18 months, where they were randomized to an additional dose of MV. Severe adverse events were monitored through surveillance of hospital admissions [10] and of mortality [8] for both outcomes the early MV group had less severe events.

### Growth

Anthropometric measurements were conducted by trained field assistants at each visit to the health center; at inclusion, 9 and 24 months (No measurements were obtained by the end of follow-up at 36 months of age). Growth was assessed by measuring length, weight and middle-upper arm circumference (MUAC). Weight of the undressed child was measured by an electronic scale (SECA 336) to the nearest 0.01 kg while the child was lying or sitting. Length was measured (SECA 416) while the child was lying down. MUAC was measured with a non-stretch TALC tape on the upper left arm in mm.

### Hypotheses and statistical analysis

All statistical analyses were conducted in STATA 11. Length and weight were converted to Z-scores using WHO 2006 standards; lenght-for-age, weight-for-age and weight-for-length [14]. MUAC was not converted to Z-score as the recommendation for MUAC cutoffs is not valid for children under 6 months of age [14, 15]. Furthermore, we have found that MUAC is as good a predictor of mortality as MUAC z-score [16].

The analysis of mortality had found no differences in mortality rates according to type of vaccine (Edmonston-Zagreb MV (Group B) or Schwarz MV (Group C)); we likewise found no difference in growth rates according to type of vaccine, and therefore combined the groups. By 18 months of age, the children in groups B and C were furthermore randomized to an additional MV or no additional MV. We found no effect of the additional MV (data not shown) and therefore combined the two groups. Thus, the present analysis focusses on the effect of receiving an early MV at 4.5 months of age (Group A) versus no early MV (Groups B and C).

We used linear regression to analyze the anthropometric outcomes at 9 and 24 months of age. All analyses were adjusted for the baseline measurement at 4.5 months of age.

Potential deviations from linearity were assessed. We constructed scatterplots of the residuals to check for normal distribution, to ensure the assumptions of the model.

We had specified in the protocol that we would stratify our analysis by sex and season. Furthermore, we stratified by NVAS as we had observed interaction between Early MV and NVAS with respect to mortality [8, 17].

We conducted a large number of tests increasing the risk of chance findings. To control for multiple testing we conducted permutation tests [18] in the main analysis and in each of the pre-specified subgroup analyses (For further descriptions see Additional file 1).

### Sample size

The study size was based on the main outcome mortality. With 6000 children and a mean MUAC of 14.55 cm (SD = 1.3 cm) by 9 months of age, we would be able to show a difference of 0.1 cm in MUAC with a power of 80% and a significance level of 0.05.

## Results

**Fig. 1:**
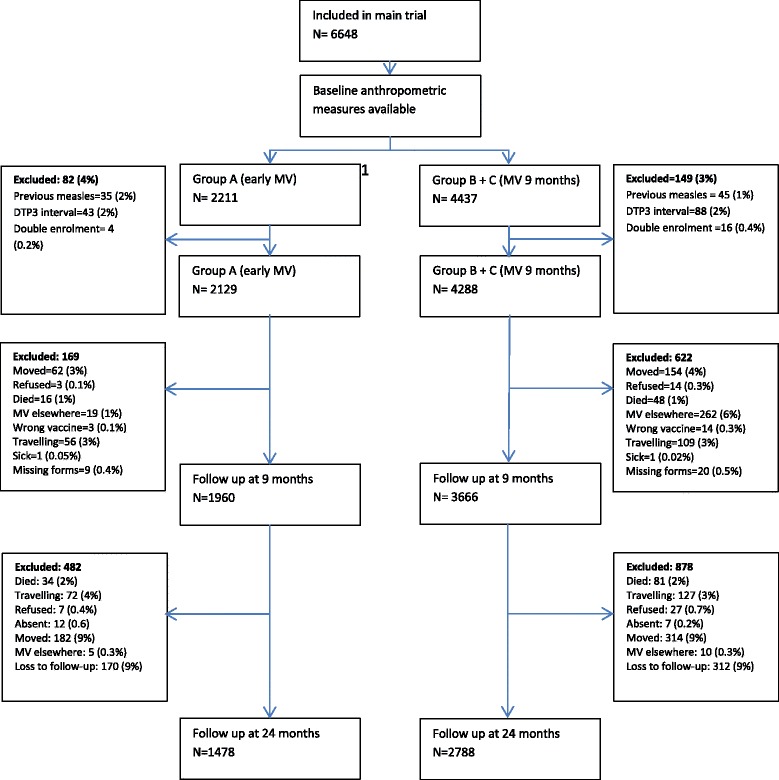
Flowchart

A total of 6648 children were enrolled in the main trial between August 2003 and April 2007 (Fig. 1). The participant flow has been described in detail elsewhere [8]; 6,417 children were randomized, 2129 to receive MV at 4.5 months and 9 months (intervention group) and 4288 were randomized to MV at 9 months of age only (control group). A total of 1960 (92%) children from the intervention group and 3666 (85%) from the control group were seen at the health center at 9 months. At 24 months, this was 1475 (69%) and 2777 (65%), respectively.

There were no differences in demographic, socioeconomic or health related background factors between the children in the two randomization groups (Additional file 2: Table S1). Demographic, socioeconomic or health related factors were also similar for children in the two groups who were lost to follow-up at 9 months and 24 months (Additional file 3: Table S2, Additional file 4: Table S3).

### Anthropometry

At 9 months of age, there were no significant differences in MUAC or in z-scores of weight-for-age and length-for-age between the two randomization groups neither overall nor when stratified on sex, season and NVAS (Table 1 and Additional file 5: Figure S1).

**Table 1:** Effect of an early MV on anthropometric measures at 9 and 24 months of age^1^

	All			Boys			Girls		
	Early MV	MV	Difference^1^ (CI: 95%)	Early MV	MV	Difference^1^ (CI: 95%)	Early MV	MV 9	Difference^1^ (CI: 95%)
	Mean (SD)	Mean (SD)		Mean (SD)	Mean (SD)		Mean (n)	Mean (n)	
ALL 9 MONTHS OF AGE									
	1960	3666		998	1826		962	1840	
WAZ (weight-for-age, z-score)	−0.31(1.19)	−0.33(1.16)	0.01(−0.03;0.04)	−0.40(1.21)	−0.34(1.19)	−0.02(−0.07;0.04)	−0.22(1.16)	−0.31(1.13)	0.03(−0.02;0.08)
LAZ (length-for-age, z-score)	−0.53(1.25)	−0.52(1.22)	−0.01(−0.06;0.04)	−0.61(1.26)	−0.61(1.24)	−0.03(−0.10;0.05)	−0.45(1.23)	−0.44(1.20)	0.00(−0.07;0.08)
MUAC (mid upper arm circumference, cm)	14.55(1.24)	14.53(1.26)	0.00(−0.04;0.05)	14.71(1.24)	14.75(1.23)	−0.01(−0.08;0.06)	14.39(1.23)	14.32(1.26)	0.01(−0.05;0.08)
ALL 24 MONTHS OF AGE									
	1478	2788		735	1394		743	1394	
WAZ (weight-for-age, z-score)	−0.68(1.02)	−0.68(1.03)	0.01(−0.04;0.06)	−0.77(1.03)	−0.71(1.06)	−0.01(−0.08;0.05)	−0.59(1.02)	−0.64(0.98)	0.03(−0.04;0.10)
LAZ (length-for-age, z-score	−1.01(1.14)	−1.01(1.13)	0.01(−0.05;0.07)	−1.12(1.14)	−1.11(1.15)	−0.02(−0.11;0.06)	−0.90(1.13)	−0.91(1.10)	0.03(−0.05;0.12)
MUAC (mid upper arm circumference, cm)	15.14(1.11)	15.06(1.13)	0.08(0.02;0.14)*	15.19(1.08)	15.18(1.15)	0.05(−0.04;0.13)	15.09(1.14)	14.94(1.09)	0.12(0.03;0.20)*

*Significant (*P* < 0.05) after controlling for multiple testing using the permutation test

^1^Statistical test was linear regression controlled for weight/length/MUAC at inclusion

**Fig. 2:**
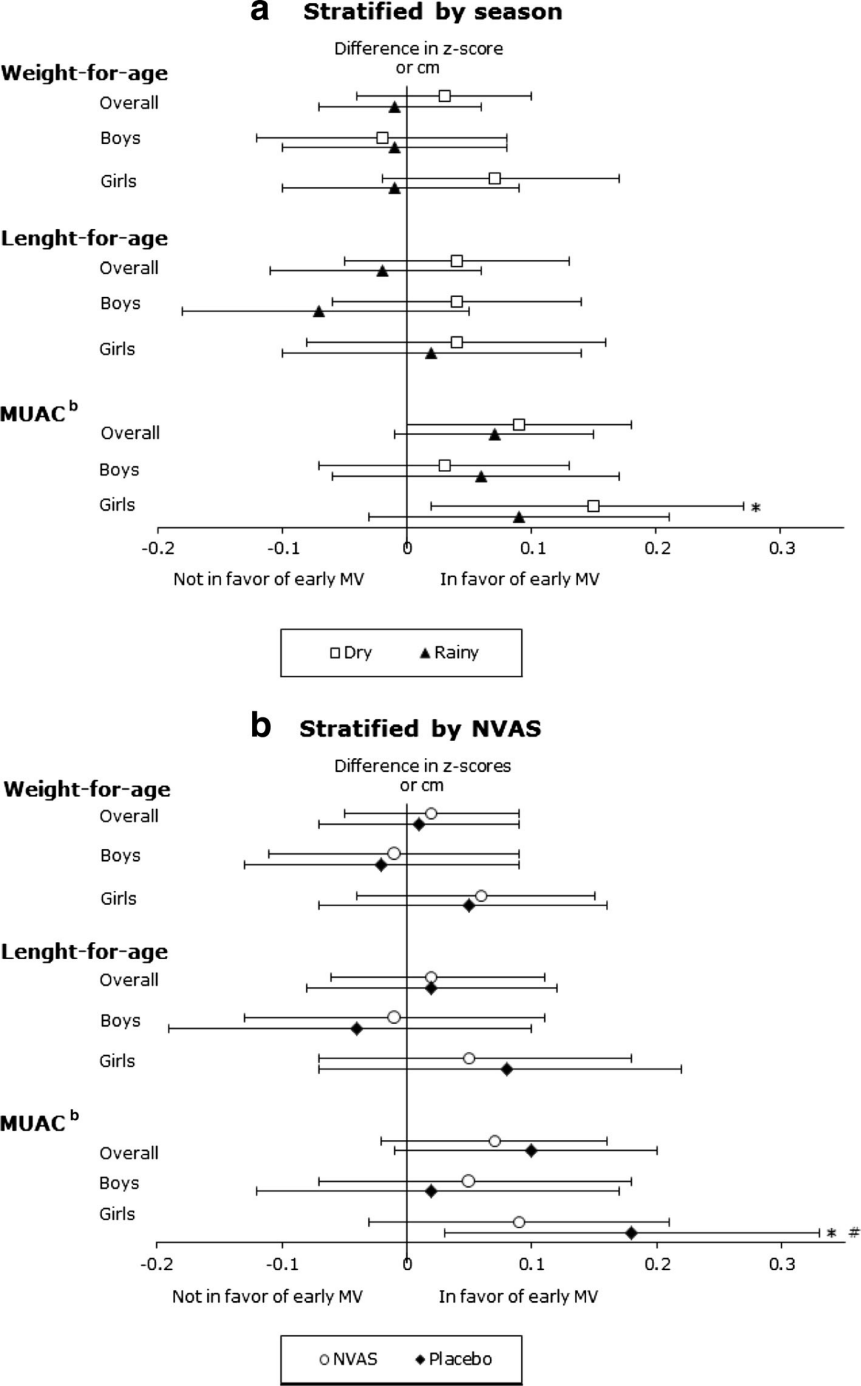
Effect of an early MV on anthropometric measures stratified by season (**a**) and NVAS (**b**) at 24 months^2^. 2) Statistical test was linear regression comparing early MV/No early MV controlled for weight/length and MUAC at inclusion. Horizontal lines defines the 95% confidence interval. *Marks significant effect of early MV (*p* < 0.05). ^b^MUAC (mid upper arm circumference). # Significant (*p* < 0.05) after controlling for multiple testing using the permutation test

At 24 months of age, early MV was associated with significantly larger MUAC overall (0.08; 95%CI:0.02;0.14). This effect was significant in girls (0.12(0.03;0.20)) but not in boys (Table 1). The effect of early MV on MUAC in girls was significant in the dry season (0.15(0.02;0.27)). Among girls who received placebo in the NVAS trial the effect of early MV on MUAC was furthermore significant (0.18(0.03;0.33)) (Fig. 2). No effects were seen on weight-for-age and length-for-age at 24 months.

When controlling for multiple testing using the permutation test, we found that the effect on MUAC at 24 months was still significantly larger in the intervention group. Furthermore, the effect on girls’ MUAC at 24 months overall and in the subgroup who did not receive NVAS at birth remained significant. However, when adjusting for multiple testing, the effect on MUAC at 24 months among girls included in the dry season was no longer significant.

## Discussion

There were no overall consistent effects of early MV on growth. However, we saw a positive effect on MUAC at 24 months of age. This effect was most pronounced among girls, especially those enrolled during the dry season and those who received placebo at birth.

### Strengths and weaknesses

The major strength of this study is the randomized design, as well as the large number of study participants. In Bissau, there is in general a large migration in and out of the study area. However at 9 months only 12% (intervention 8%; control 14%) were lost to follow up (FU), but there was an imbalance when comparing the two randomization groups, as more children in the control group had received MV elsewhere and were therefore excluded from the analysis, because the time of vaccine, vaccine strain and quality of the MV was unknown. Assuming that these unknown MV vaccinations in the 9 months MV group also had a beneficial effect, they would have lowered the difference in MUAC and reduced the estimated beneficial effect of early MV between 4.5 and 24 months of age. However among children excluded there were no baseline differences. At 24 months 21% were lost to FU (intervention 23%, control 20%), mainly because they were travelling or had moved during the study period. Furthermore there were no baseline differences between the groups of children lost to FU. Nonetheless, it clearly reduced our power, because of an expected 10% lost to follow-up.

The staff performing the health examination and growth measurements were blind with regard to randomization allocation. All staff were trained in performing the measurements according to best practice and the health examinations were conducted by a trained pediatrician. For ethical reasons the mother was not blinded with regard to randomization allocation, but the staff assessing outcome was blinded, and we find it unlikely that this would have influenced the outcome assessment on growth.

When conducting a large number of tests there is always a risk of chance findings. Therefore, we also conducted permutation tests. We found that MUAC at 24 months overall as well as for girls and for children who had received placebo in the NVAS trial remained significantly larger in the early MV group after controlling for multiple testing.

### Consistency with other studies

To our knowledge this study is the first to assess the effect of an early, additional dose of MV on growth. Although we did not find any consistent effects of an early MV with growth, we did see a beneficial effect on MUAC at 24 months, especially among girls receiving early MV in the dry season and among those who received placebo NVAS.

MUAC is a valid measure of nutritional status and a good predictor of mortality [16, 19]. The finding that early MV had a positive effect on MUAC among girls at 24 months corresponds with previous studies from the same trial concluding that the effects of early MV were strongest among girls. Thus, mortality and hospital admission rates were significantly lower among girls [8, 10]. Significantly larger MUAC was seen in relation to dry season and girls, and in relation to girls and placebo NVAS at 24 months.

However, the lower mortality in the intervention group may have biased the later comparisons of growth. Because those children who died in the control group may have had poorer growth, which would have caused a greater difference in the compared outcomes between the intervention group and the control group if they had survived. Nevertheless, as we adjusted all analyses for baseline growth parameters this bias may only have had limited effect on the results.

### Interpretation

It is possible that an early MV can lead to better nutritional status (higher MUAC) and hereby explain the reduction in mortality seen. However, as the children generally were not malnourished (MUAC at enrollment, mean: 14.2 cm, SD (12)), this does not seem likely. Rather, children who experience repeated infections fail to thrive and children with repeated infections generally have poorer nutritional status [20, 21]. Hence, the higher MUAC is probably a result of less infection in the intervention group. The reason why MUAC is the only parameter affected, could be that it is a more sensitive measure compared to e.g., length, why small changes can be observed [22]. It is also known that MUAC is just as good or even better way of predicting mortality [23]. We have previously reported that NVAS at birth may interact with vaccines given later [8, 24]. In regard to early MV we found that NVAS abrogated the beneficial effect of early MV on mortality and this may also be the case for growth. However, the difference in MUAC between the two groups was relatively small (around 0.1 cm) and is seen only at 24 months, and the question is, if this makes a big difference in relation to health. It is likely to confirm that an early MV to a lesser extent influences growth. We have previously observed more beneficial effects of MV in females and in the dry season. We have no good explanation for this finding but speculate that MV may protect against certain seasonal pathogens, which may be more frequent in females, explaining why girls receiving early MV in the dry season have better growth than girls receiving early MV in the rainy season.

## Conclusion

According to current understanding, a vaccine only protects against a specific pathogen. The findings that an early additional MV is associated with lower mortality and lower risk of hospital admission against non-measles related infections, especially respiratory infections, questions this paradigm and has pointed to a new understanding of vaccines. The evidence for non-specific effects of vaccines has recently been reviewed by WHO [25] and it was concluded that more research is needed, also into the potential biological mechanisms behind the non-specific effects. This study suggests that the non-specific effects of the MV are mainly seen on mortality and hospital admissions and to a lesser extent or not at all on growth.

## Additional files

Additional file 1: Multiple testing. An explanation of the permutation test conducted to control for multiple testing. (DOCX 14 kb)
Additional file 2: Table S1. Baseline characteristics by randomization group. Baseline characteristics at 4.5 months by the two randomization groups, one receiving early MV and MV at 9 months, the other receiving only MV at 9 months. There were no differences in demographic, socioeconomic or health related background factors between the two randomization groups. (DOCX 17 kb)
Additional file 3: Table S2. Baseline characteristics by randomization group in those excluded at 9 months. Baseline characteristics at 4.5 months by randomization group among children excluded at 9 months of age. There were no differences in demographic, socioeconomic or health related background factors between the excluded children in the two randomization groups. (DOCX 18 kb)
Additional file 4:
**Table S3.** Baseline characteristics by randomization group in those lost to follow-up by 24 months. Baseline characteristics at 4.5 months by randomization group among children lost to follow-up by 24 months of age. There were no differences in demographic, socioeconomic or health related background factors between the children low to follow-up in the two randomization groups. (DOCX 18 kb)
Additional file 5:
**Figure S1.** Effect of an early MV on anthropometric measures stratified by season or NVAS at 9 months^1.^ Legend: 1) Statistical test was linear regression comparing early MV/No early MV controlled for weight/length and MUAC at inclusion. Horizontal lines defines the 95% confidence interval. *Marks significant effect of early MV (p < 0.05). ^a^MUAC (mid upper arm circumference). A figure of the effect of an early MV on weight-for-age, length-for-age and mid-upper-arm-circumference at 9 months stratified by season or NVAS. (PDF 99 kb)

## Abbreviations

MV: Measles vaccine
NVAS: Neonatal vitamin A supplementation
MUAC: Mid-upper-arm circumference
WHO: World Health Organization
MA: Maternal antibodies
MRR: Mortality rate ratio
BHP: Bandim Health Project
HDSS: Health and demographic surveillance system
OPV: Oral polio vaccine
DTP: Diphtheria, tetanus, pertussis
WAZ: Weight-for-age z-score
LAZ: Length-for-age z-score
FU: Follow up
SD: Standard deviation
