# Supplementary material for: The effect of early measles vaccination at 4.5 months of age on growth at 9 and 24 months of age in a randomized trial in Guinea-Bissau
Source: BMC Pediatr. 2016 Dec 3;16:199. doi: 10.1186/s12887-016-0738-z (PMC5135799; doi:10.1186/s12887-016-0738-z)
Supplement: Additional file 1: — Multiple testing. An explanation of the permutation test conducted to control for multiple testing. (DOCX 14 kb) [file 12887_2016_738_MOESM1_ESM.docx]

*Multiple testing*

The concept of the permutation test is: if there is no experimental effect, then the labelling of observations as “treated” and “untreated” is arbitrary, the same data would arise with any other labelling. Relabeling or permuting the labels gives a sampling null-distribution of the maximum statistic which the observed maximum statistic can be compared with. As test statistics we used the absolute Wald-statistic from the linear regressions on continuous outcomes. We randomly permuted the randomization group label 1000 times, each time obtaining the maximum of the seven individual absolute Wald-statistics. We used the 95% largest of these 1000 maximum statistics as the critical value; the observed maximum statistic was deemed significant and the joint null hypothesis was rejected if it was larger than this critical value. The joint null hypothesis was defined as no MV effect on all outcomes ([16](#_ENREF_16)).
