# Supplementary material for: The effect of early measles vaccination at 4.5 months of age on growth at 9 and 24 months of age in a randomized trial in Guinea-Bissau
Source: BMC Pediatr. 2016 Dec 3;16:199. doi: 10.1186/s12887-016-0738-z (PMC5135799; doi:10.1186/s12887-016-0738-z)
Supplement: Additional file 2: Table S1. — Baseline characteristics by randomization group. Baseline characteristics at 4.5 months by the two randomization groups, one receiving early MV and MV at 9 months, the other receiving only MV at 9 months. There were no differences in demographic, socioeconomic or health related background factors between the two randomization groups. (DOCX 17 kb) [file 12887_2016_738_MOESM2_ESM.docx]

## Baseline characteristics by randomization group

|  | **Main analysis** | |
| --- | --- | --- |
|  | **Early MV (N=2129)** | **No early MV (N=4288)** |
| **Demographic factors** | | |
| Child age at enrolment; months. (Interquartil range) | 4.8 (4.7-5.2) | 4.8 (4.7-5.2) |
| Bandim district; % (n) | 42 (898) | 42 (1794) |
| Female; % (n) | 49 (1045) | 50 (2137) |
| **Socio-economic factors** | | |
| Number of people/bed | 2.9 | 2.9 |
| No people/room | 4.1 | 4.2 |
| House has toilet; % (n) | 16 (336) | 15 (630) |
| House has functioning electricity; % (n)* | 36 (535) | 37 (1108) |
| **Anthropometry** | | |
| Mean child weight; kg. (SD) | 7.16 (0.98) | 7.14 (0.99) |
| Mean child MUAC^a^; mm. (SD) | 142 (12) | 141 (12) |
| Mean child height; cm. (SD) | 64 (2.8) | 64 (2.8) |
| Maternal MUAC^a^; mm. (SD) | 275 (34) | 274 (34) |
| **Z-scores** | | |
| Child weight; z, mean (SD) | -0.10(1.14) | -0.12(1.13) |
| Child height; z, mean (SD) | -0.38(1.20) | -0.39(1.22) |
| **Health status** | | |
| Reported fever; % (n) | 8 (175) | 9 (388) |
| Diarrhea; % (n) | 5 (103) | 4 (186) |
| Temp>37.5 ; % (n) | 5 (30) | 5 (59) |
| Respiratory rate; per minute (SD) | 42 (5.0) | 43 (5.2) |
| Eye problems; % (n) | 0.5 (11) | 0.4 (16) |
| Skin infection; % (n) | 1.1 (24) | 1.6 (69) |
| Respiratory infection; % (n) | 7 (147) | 7 (302) |
| **Vitamin A at birth** | | |
| First vitamin A trial; % (n) | 34 (719) | 34 (1466) |
| Second vitamin A trial; % (n) | 44 (944) | 44 (1883) |
| **Season** | | |
| Dry season; % (n) | 50 (1063) | 50 (2149) |

^a^MUAC=Mid-upper-arm-circumference.
*Of those with information (N=1492 in the early MV group, N=3015 in the no early MV group)
