# Supplementary material for: The effect of early measles vaccination at 4.5 months of age on growth at 9 and 24 months of age in a randomized trial in Guinea-Bissau
Source: BMC Pediatr. 2016 Dec 3;16:199. doi: 10.1186/s12887-016-0738-z (PMC5135799; doi:10.1186/s12887-016-0738-z)
Supplement: Additional file 3: Table S2. — Baseline characteristics by randomization group in those excluded at 9 months. Baseline characteristics at 4.5 months by randomization group among children excluded at 9 months of age. There were no differences in demographic, socioeconomic or health related background factors between the excluded children in the two randomization groups. (DOCX 18 kb) [file 12887_2016_738_MOESM3_ESM.docx]

## Baseline characteristics by randomization group in those excluded at 9 months

|  | **Exclusion analyses at 9 months** | |  |
| --- | --- | --- | --- |
|  | **Early MV**  **N=169** | **No early MV**  **N=622** |  |
| **Demographic factors** | | | **P-value** |
| Child age at enrolment; months. (Interquartil range) | 4.9 (4.7-5.2) | 4.9 (4.7-5.2) | 0.53 |
| Bandim district; % (n) | 44 (74) | 36 (225) | 0.07 |
| Female sex; % (n) | 49 (83) | 48 (297) | 0.75 |
| **Socio-economic factors** | | | |
| No people/bed | 2.9 | 2.9 | 0.40 |
| No people/room | 4.0 | 4.2 | 0.23 |
| House has toilet; % (n) | 11 (19) | 15 (92) | 0.24 |
| House has functioning electricity*; % (n) | 35 (44) | 39 (167) | 0.50 |
| **Anthropometry** | | | |
| Child MUAC^a^; cm. mean (SD) | 14 (1.22) | 14 (1.27) | 0.18 |
| Child weight; z, mean (SD) | -0.30(1.28) | -0.27(1.19) | 0.77 |
| Child height; z, mean (SD) | -0.54(1.16) | -0.53(1.14) | 0.90 |
| Maternal MUAC^a^; mm. Mean (SD) | 27 (3.25) | 27 (3.17) | 0.33 |
| **Health status** | | |  |
| Reported fever; % (n) | 7 (12) | 11 (68) | 0.13 |
| Diarrhea; % (n) | 4 (6) | 5 (31) | 0.43 |
| Clinical fever; % (n) | 1 (1) | 2 (14) | 0.32 |
| Respiratory rate; per minute (SD) | 42 (4.27) | 43 (5.24) | 0.13 |
| Skin infection; % (n) | 3 (5) | 2 (11) | 0.32 |
| Respiratory infection; % (n) | 5 (9) | 8 (47) | 0.31 |
| **Vitamin A at birth** | | |  |
| First vitamin A trial | 34 (57) | 35 (219) | 0.72 |
| Second vitamin A trial | 39 (66) | 35 (220) | 0.38 |
| **Season** | | | |
| Dry season | 53 (90) | 50 (311) | 0.45 |

^a^MUAC=Mid-upper-arm-circumference.
*Of those with information (N=124 in the early MV group, N=430 in the no early MV group)
