# Supplementary material for: The effect of early measles vaccination at 4.5 months of age on growth at 9 and 24 months of age in a randomized trial in Guinea-Bissau
Source: BMC Pediatr. 2016 Dec 3;16:199. doi: 10.1186/s12887-016-0738-z (PMC5135799; doi:10.1186/s12887-016-0738-z)
Supplement: Additional file 4: — Table S3. Baseline characteristics by randomization group in those lost to follow-up by 24 months. Baseline characteristics at 4.5 months by randomization group among children lost to follow-up by 24 months of age. There were no differences in demographic, socioeconomic or health related background factors between the children low to follow-up in the two randomization groups. (DOCX 18 kb) [file 12887_2016_738_MOESM4_ESM.docx]

## Baseline characteristics by randomization group in those excluded at 24 months

|  | **Exclusion analyses 24 months** | |  |
| --- | --- | --- | --- |
|  | **Early MV**  **N= 482** | **No early MV**  **N=878** |  |
| **Demographic factors** | | | **P-value** |
| Child age at enrolment; months. (Interquartil range) | 4.9 (4.7-5.2) | 4.8 (4.7-5.2) | 0.90 |
| Bandim district; % (n) | 40 (194) | 41 (362) | 0.72 |
| Female sex; % (n) | 45 (219) | 51 (446) | 0.06 |
| **Socio-economic factors** | | | |
| No of people/bed | 2.9 | 2.9 | 0.85 |
| No people/room | 4.7 | 4.8 | 0.70 |
| House with toilet; % (n) | 16 (79) | 14 (126) | 0.40 |
| House has functioning electricity *; % (n) | 30 (110) | 31 (213) | 0.77 |
| **Anthropometry** | | | |
| Child MUAC^a^; cm. mean (SD) | 14 (1.21) | 14 (1.21) | 0.17 |
| Child weight; z, mean (SD) | -0.04 (1.20) | -0.14 (1.16) | 0.15 |
| Child height; z, mean (SD) | -0.39 (1.19) | -0.45 (1.14) | 0.43 |
| Maternal MUAC^a^; cm. Mean (SD) | 27 (3.36) | 27 (3.46) | 0.74 |
| **Health status** | | |  |
| Reported fever; % (n) | 9 (45) | 8 (83) | 0.96 |
| Diarrhea, ; % (n) | 5 (24) | 6 (51) | 0.53 |
| Clinical fever; % (n) | 2 (11) | 1 (13) | 0.43 |
| Respiratory rate; per minute | 42 (4.78) | 43 (5.18) | 0.50 |
| Eye problems (yes) ; % (n) | 1 (3) | 0.35 (3) | 0.46 |
| Skin infection; % (n) | 1 (6) | 2 (19) | 0.22 |
| Respiratory infection; % (n) | 8 (38) | 8 (71) | 0.87 |
| **Vitamin A at birth** | | | |
| First vitamin A trial | 23 (111) | 24 (208) | 0.78 |
| Second vitamin A trial | 53 (256) | 52 (456) | 0.68 |
| **Season** | | | |
| Dry season | 61 (294) | 61 (534) | 0.95 |

^a^MUAC=Mid-upper-arm-circumference.
*Of those with information (N=361 in the early MV group, N=679 in the no early MV group)
