# Supplementary figures and images for: The effect of early measles vaccination at 4.5 months of age on growth at 9 and 24 months of age in a randomized trial in Guinea-Bissau
Source: BMC Pediatr. 2016 Dec 3;16:199. doi: 10.1186/s12887-016-0738-z (PMC5135799; doi:10.1186/s12887-016-0738-z)

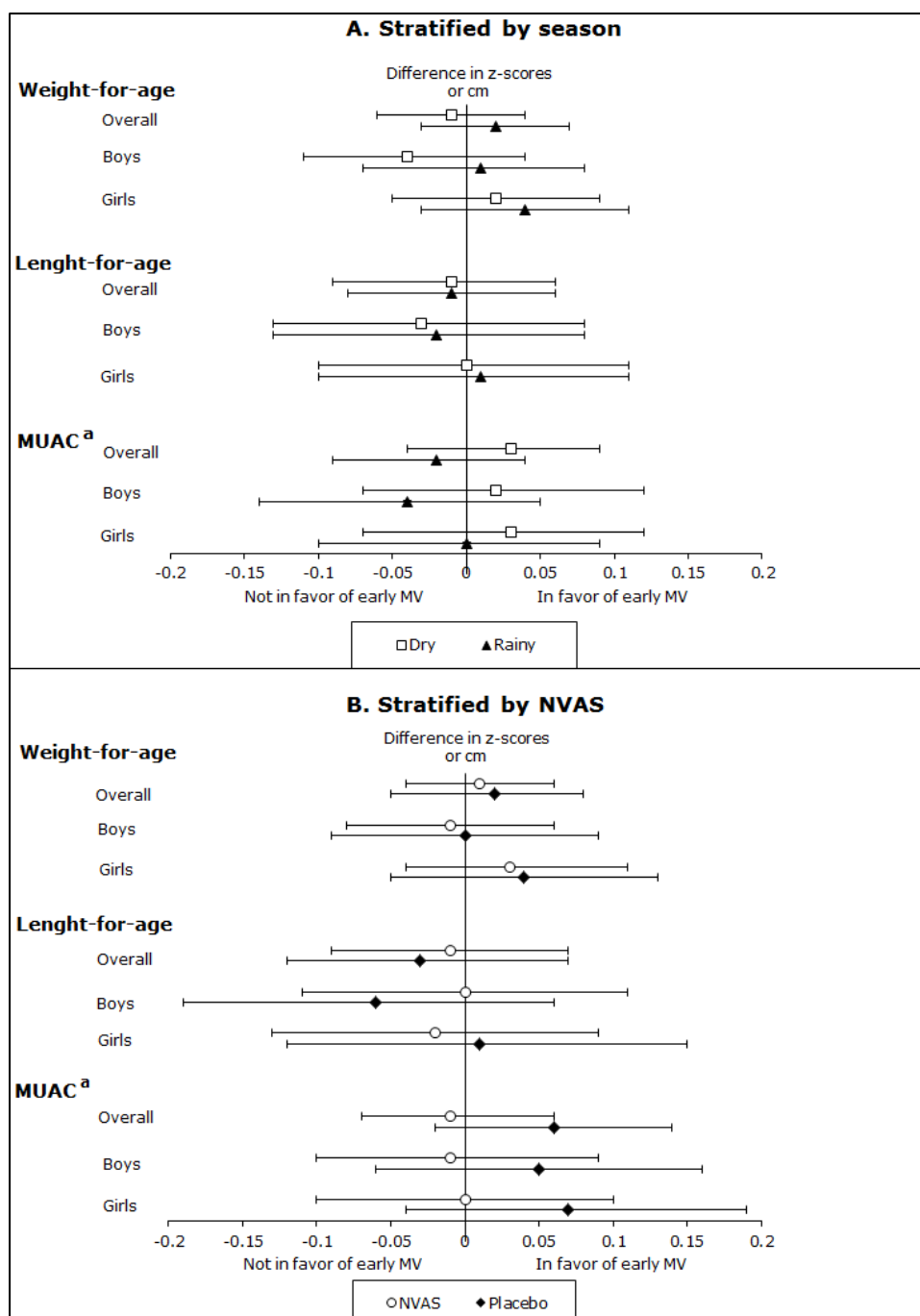

Supplement: Additional file 5: — Figure S1. Effect of an early MV on anthropometric measures stratified by season or NVAS at 9 months1. Legend: 1) Statistical test was linear regression comparing early MV/No early MV controlled for weight/length and MUAC at inclusion. Horizontal lines defines the 95% confidence interval. *Marks significant effect of early MV (p < 0.05). aMUAC (mid upper arm circumference). A figure of the effect of an early MV on weight-for-age, length-for-age and mid-upper-arm-circumference at 9 months stratified by season or NVAS. (PDF 99 kb) [file 12887_2016_738_MOESM5_ESM.pdf]
